# Tracking the early signals of crude oil in seawater and plankton after a major oil spill in the Red Sea

**DOI:** 10.1007/s11356-023-27111-0

**Published:** 2023-05-03

**Authors:** Sreejith Kottuparambil, Ananya Ashok, Alan Barozzi, Grégoire Michoud, Chunzhi Cai, Daniele Daffonchio, Carlos M. Duarte, Susana Agusti

**Affiliations:** 1 Red Sea Research Center (RSRC), King Abdullah University of Science and Technology (KAUST), Thuwal, Saudi Arabia; 2 Computational Bioscience Research Center (CBRC), King Abdullah University of Science and Technology, Thuwal, Saudi Arabia

**Keywords:** Red Sea, Oil spill, PAHs, CDOM, MAGs, δ^13^C, LNA bacteria

## Abstract

Understanding the immediate impacts of oil spills is essential to recognizing their long-term consequences on the marine environment. In this study, we traced the early (within one week) signals of crude oil in seawater and plankton after a major oil spill in October 2019 in the Red Sea. At the time of sampling, the plume had moved eastward, but we detected significant signs of incorporation of oil carbon into the dissolved organic carbon pool, resulting in a 10–20% increase in the ultraviolet (UV) absorption coefficient (a_254_) of chromophoric dissolved organic matter (CDOM), elevated oil fluorescence emissions, and depletion of the carbon isotope composition (δ^13^C) of the seawater. The abundance of the picophytoplankton *Synechococcus* was not affected, but the proportion of low nucleic acid (LNA) bacteria was significantly higher. Moreover, specific bacterial genera (*Alcanivorax*, *Salinisphaera*, and *Oleibacter*) were enriched in the seawater microbiome. Metagenome-assembled genomes (MAGs) suggested that such bacteria presented pathways for growing on oil hydrocarbons. Traces of polycyclic aromatic hydrocarbons (PAHs) were also detected in zooplankton tissues, revealing the rapid entry of oil pollutants into the pelagic food web. Our study emphasizes the early signs of short-lived spills as an important aspect of the prediction of long-term impacts of marine oil spills.

**Supplementary Information:**

The online version contains supplementary material available at 10.1007/s11356-023-27111-0.

## Introduction

Petrochemical compounds released by accidental oil spills are a globally growing concern for oceans due to their deleterious impacts on ecosystems and resources. In the marine environment, major sources of oil spills are operational discharges from marine vessels, unintended spills from tankers, and accidents related to oil drilling platforms (Zhang et al. 2019). Crude petroleum oil is a complex mixture of over 17000 identified chemicals where aromatic hydrocarbons and their derivatives represent most of their non-polar fractions (Daghio et al. 2017; Head et al. 2006). The saturated hydrocarbons in crude oil are biodegradable; however, high molecular weight polycyclic aromatic hydrocarbons (PAHs) and the polar fractions are more persistent and toxic to the environment (Head et al. 2006). In addition, oil spills are prevalent sources of heavy metal contamination in affected areas (Zhang et al. 2020).

Various degradation processes of crude oil on the sea surface lead to the incorporation of dispersed components into the marine food web, mainly through planktonic organisms (Chanton et al. 2012). Bioaccumulation of petroleum hydrocarbons at the base of the marine food web could subsequently elevate the degree of exposure of higher organisms to oil-derived toxic compounds (Wilson et al. 2016). The ecological impacts of oil spills are widespread among marine organisms including reduced growth/reproduction, increased mortality, and impairment of the physiology of plankton, invertebrates, fishes, and mammals (Zhang et al. 2019). Bioaccumulation of crude oil lipid-soluble fractions in marine organisms is another concern (Chase et al. 2013; Noh et al. 2018; Yu et al. 2019). Moreover, seafood contamination with carcinogenic PAHs causes serious human health concerns (Farrington 2020). Thus, besides the impacts of oil spills on coastal resources, aquaculture, and tourism, their negative effects on wildlife can be long-lasting.

Marine planktonic organisms play a significant role in determining the fate of oil-derived persistent organic pollutants (POPs) in oceans such as heavy metals and PAHs. The carbon from subsurface oil slicks gets incorporated into the marine food chain, primarily through planktonic organisms (Graham et al. 2010). Besides the direct and indirect toxic impacts of oil hydrocarbons on individuals, planktonic organisms significantly contribute to the flux of oil from the surface to the bottom layers and their sedimentation to the seafloor. While accumulation in phytoplankton is the key entry pathway for lipophilic organic pollutants such as PAHs in marine food chains (Ashok et al. 2020; Del Vento & Dachs 2002), zooplankton provides important sinks and vectors for their transport in the marine environment by egestion through sinking fecal pellets and decaying biomass (Berrojalbiz et al. 2009). Moreover, they transfer oil hydrocarbons to higher trophic levels in the oceanic food web through cellular ingestion and subsequent predation (Daly et al. 2021). The adverse effects of oil spills on marine organisms vary with the nature and volume of the spilled oil and the sensitivity of the biota affected (Li et al. 2016).

Significant advancements have been made in marine oil spill preparedness and countermeasures in recent decades. These include the development of earth observation–based systems for early warning and real-time monitoring (Blondeau-Patissier et al. 2023; Dong et al. 2022), effective spill response strategies (Ivshina et al. 2015; Li et al. 2016), and efficient clean-up actions (Dave & Ghaly 2011). The primary step in oil spill risk assessment is the identification of potential adverse impacts caused by the contaminant or by the response actions taken (Chen et al. 2019). Within the initial few hours of an oil spill, petroleum hydrocarbons partition rapidly into the air and seawater (Gros et al. 2014) resulting in elevated levels of hydrocarbons in the surface water. This increases the risk of biological exposure in the euphotic zone during the first few days, which can be particularly detrimental to marine biota, especially planktonic organisms (Brussaard et al. 2016). Therefore, it is vital to assess the immediate impacts of oil spills. However, due to the accidental nature of the event and usual delays in response actions, the early impacts often remain unnoticed.

The Red Sea is an area with intense maritime shipping activities including heavy traffic of oil tankers (Periáñez 2020), with an estimated average daily transport of one million barrels of oil along its main axis (Kleinhaus et al. 2020b). Consequently, the Red Sea basin is at high risk of accidental oil spills of varying magnitudes. The unique water current dynamics along the Red Sea cause the cycling and redistribution of hydrocarbon pollutants (Kostianaia et al. 2020) and contamination of coastal resources and pristine ecosystems such as coral reefs. Although Red Sea organisms show resilience to oil contaminants (Alwakeel 2017; Kottuparambil & Agusti 2018), imminent oil spills are potential threats to the diversity and ecological health of unique Red Sea ecosystems (Kleinhaus et al. 2020b). Despite being a high-risk area for oil pollution and a high frequency of oil spills in recent history, comprehensive studies about the fate and ecological consequences of oil spills in the Red Sea are rare.

The goal of the present study is to examine the changes in water quality and the accumulation of oil-derived persistent pollutants in the water and zooplankton nearly after the oil spill occurred due to an explosion of the Iranian oil tanker Sabiti on 11th of October 2019 in the central Red Sea (Nukapothula et al. 2021). After the explosion, the tanker moved toward the south, leaving a thick surface plume of crude oil with an estimated total length of 650 nautical miles (nm) as revealed by the Copernicus Sentinel-1B satellite images (available at http://www.beyond-eocenter.eu), indicating potential severe contamination of the surrounding sea. We collected samples four days after the spill event at three stations located at different distances from the tanker position to track the signals of the oil spill in the seawater and in the planktonic organisms, including the microbiota and the zooplankton. Even though only disperse small surface patches of crude oil were visible in the sampling area closer to the plume, our data indicated traces of crude oil in the water and pelagic organisms.

## Materials and methods

### Study site and sample collection

**Fig. 1:**
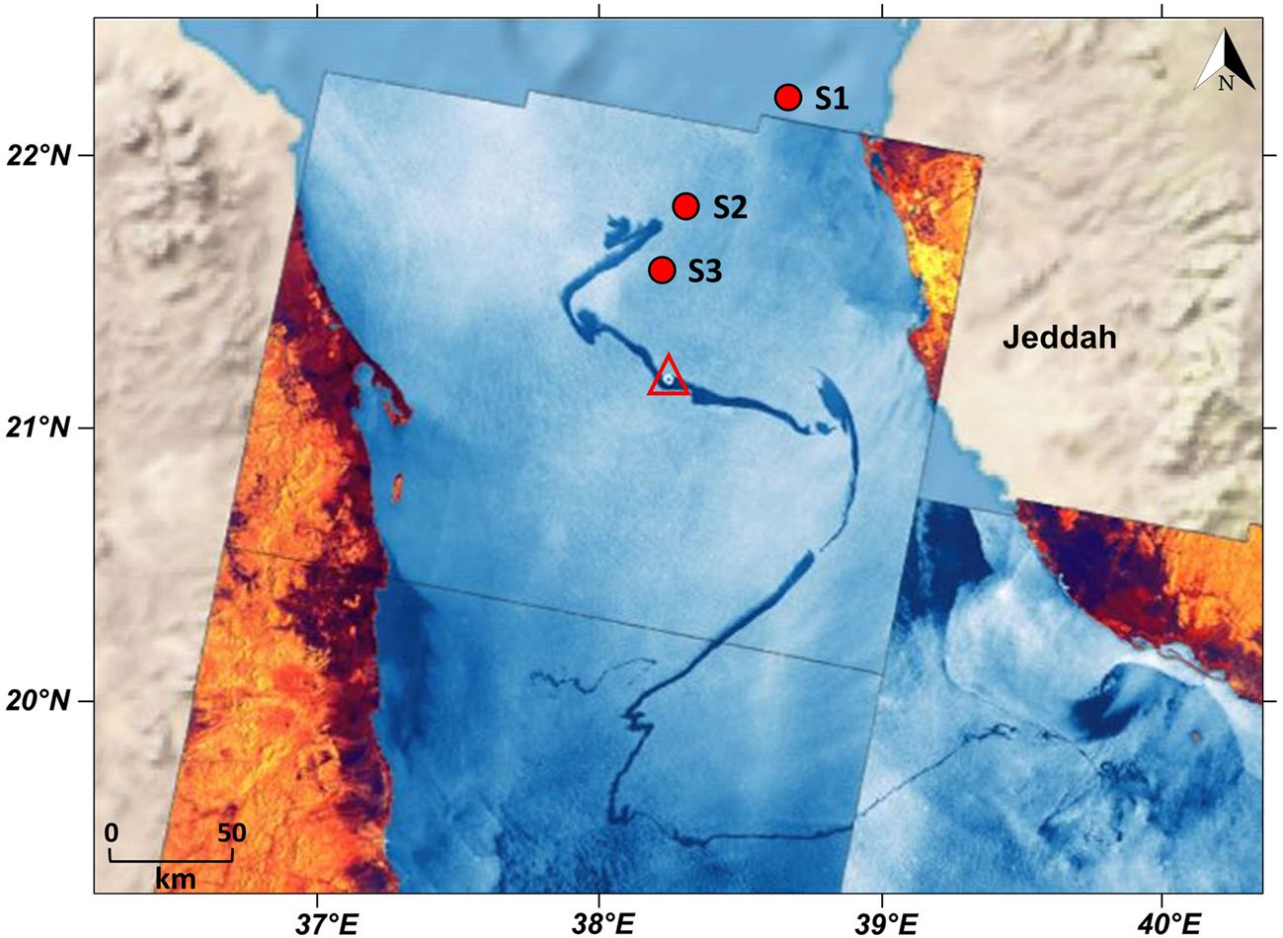
Map showing the extensive oil slick and the sampling stations after the 2019 spill in the Red Sea. The position of the damaged tanker during the event is represented by the red triangle. The map was reconstructed from Copernicus Sentinel-1 (Copernicus Program, European Union) images acquired in interferometric wide swath (IW) mode on 13 and 14 October 2019

An extensive oil spill in the Red Sea was caused by the damaged Iranian-owned oil tanker Sabiti on 11th October 2019. The accident occurred at about 55 nm off the Saudi Arabian port city of Jeddah at the point with coordinates 21.13°N and 38.33°E (Periáñez 2020), resulting in a lengthy surface oil plume spanning several hundreds of kilometers. Water and zooplankton samples were collected at three locations with varying degrees of potential exposure to oil from the source tanker in the Red Sea on 15th October (Fig. 1). Three stations were sampled: station S1 (22.11°N, 38.64°E) was located northeast of the original spill site, at a distance of about 60 nm from the source tanker during the disaster on the 13th of October 2019. Two stations where potential contamination of the surface layer likely occurred (S2 and S3 at 21.75°N, 38.27°E and 21.61°N, 38.19°E, at 35 nm and 27 nm away from the position of the tanker, respectively) were selected in the vicinity of the spill as indicated by the Copernicus Sentinel-1 satellite images from 13 to 14 October 2019 (Fig. 1).

We collected surface seawater samples from the upper photic zone to measure the immediate impacts of the oil spill. At the ambient station, we used a Niskin bottle equipped with conductivity, temperature, and depth sensors (CTD; Seabird 9+) to collect the seawater sample at a depth of 0.5 m. The collection of surface samples at stations closer to the oil spill was done manually to avoid potential oil damage to the CTD sensors. Seawater samples for total organic carbon (TOC) analysis were collected in acid-cleaned, pre-combusted 40-mL glass tubes. The acidified samples (85% H_3_PO_4_ at 0.2% v/v) were refrigerated at 4 °C in the dark until analysis. Water samples for optical analysis were collected in 250 mL amber glass bottles and stored at 4 °C. For PAH analysis, samples were collected in 250 mL amber glass bottles and preserved in 10% methanol. Seawater samples for elemental analysis were collected in 250 mL plastic bottles and immediately preserved in 1% HNO_3_. The samples for PAH analysis were immediately frozen at −20 °C. One liter of water samples was collected in acid-cleaned glass bottles for CO_2_, CH_4_, and isotopic composition analyses. The samples were stored at 4 °C.

For microbial analysis, 3 replicates of 10 L of surface seawater were collected at all the stations and filtered through 0.2-μm sterile polyethersulfone (PES) Sterivex filters (Merck Millipore, USA), using peristaltic pumps. Each filter was stored in 2-mL cryo-vials, filled with lysis buffer (sucrose 0.75 mol L^−1^, Tris-HCl 50 mmol L^−1^ pH 8.0, EDTA 40 mmol L^−1^ pH 8.0), flash-frozen in liquid nitrogen, and stored at −80 °C until further analysis. For flow cytometry (FCM) microbial analysis, 2 mL seawater samples were collected in cryo-vials and fixed with 80 μL of glutaraldehyde (25% v/v). Samples were flash-frozen in liquid nitrogen and stored at −80 °C. All samples were analyzed within one week of collection.

Zooplankton samples at the sampling sites were collected using the WP2 net (200-μm mesh, 1 m mouth diameter; KC Denmark A/S, Denmark) from 200 m depth to the surface. The collected zooplankton samples were flushed with clean seawater and transferred to a 100-μm mesh. The adhered seawater to the samples was washed away by repeated rinsing with Milli-Q distilled water. The washed samples were collected in 50-mL plastic centrifuge tubes (VWR, Radnor, PA, USA) for heavy metal estimation and 50-mL amber glass bottles for PAH analysis. All samples were preserved at −20 °C in the dark until returned to the laboratory for further analyses.

### DNA extraction and metagenomic library preparation

Total DNA extraction from seawater samples for 16S rRNA metagenomic sequencing was performed using the phenol–chloroform protocol (Green & Sambrook 2017) as described in Supplementary Method S1. DNA was quantified with a Qubit® 3.0 Fluorometer using the Qubit® dsDNA HS Assay Kits (Thermo Fisher Scientific), and DNA quality was assessed by gel electrophoresis with 1% agarose. The extracted DNA was stored at −20 °C until library preparation.

16S rRNA libraries were prepared using the Illumina® Nextera XT Sample Prep Kit, following the Illumina® 16S rRNA metagenomic sequencing library preparation protocol (Supplementary Method S1). The number of reads obtained from the 16S rDNA amplicon sequencing ranged between 90,000 and 150,000 reads per sample (Supplementary materials Fig. S1). Rarefaction curve for 16S amplicon sequencing for three surface water samples in the Red Sea is provided in Supplementary materials Fig. S2.

### Genome analysis

After quality check and trimming with Trim_galore v0.6.6 (Krueger 2018), all metagenomics reads were co-assembled into contigs with MEGAHIT v1.2.9 (Li et al. 2015) with default parameters and a minimum contig length of 1000 bp. Then, metagenome-assembled genomes (MAGs) were recovered following a modified version of the protocol used by Michoud et al. (Michoud et al. 2021) as described in Supplementary Method S2. MAGs with completeness higher than 70% and contamination lower than 10% were selected. DESeq2 (Love et al. 2014) was used to assess significantly differentially enriched MAGs in communities across the sampling stations. Raw sequencing data and the MAGs are available at NCBI under the BioProject accession PRJNA941206.

### Spectroscopic and chemical analyses of oil signals in seawater

The UV-visible absorption spectra of chromophoric dissolved organic matter (CDOM) were measured in filtered samples (0.2-μm PTFE membrane filters; Pall Life Sciences, Ann Arbor, MI, USA) using a UV-Vis spectrophotometer (Lambda 1050, PerkinElmer, Waltham, MA, USA). The CDOM spectra were recorded immediately after filtration in 10-cm path-length quartz cuvettes with a measurement range of 250–750 nm with 1-nm intervals. Filtered fresh Milli-Q water was measured as blank. All absorbance data points were corrected to the average absorbance from 600 to 750 nm to nullify the residual scattering properties of the sample. Then, the absorbance values were transformed to Naperian absorption coefficient a_λ_ (m^−1^) using the equation provided by Iuculano et al. (Iuculano et al. 2019). The variability and quality of CDOM were assessed by two proxy parameters, namely, absorption coefficients at 254 nm spectral slope over 275–295 nm (a_254_ and S_275–295_, respectively). a_254_ is proportional to the number of conjugated carbon double bonds and a proxy to the bulk dissolved organic carbon (DOC) concentration (Iuculano et al. 2019). S_275–295_, obtained by linear regression of the natural log-transformed a_λ_ spectra, is a proxy for the structural characterization of CDOM.

The fluorescence signals of light/refined and heavy oil components were estimated using a fluorescence spectrometer (LS55, PerkinElmer, Waltham, MA, USA) over excitation/emission pairs at 254/350 nm and 350/410–550 nm, respectively (Lambert 2003). All measurements were conducted at room temperature with a quartz cuvette of 1 cm path length. The instrument was pre-calibrated with a quinine sulfate standard solution (Zhou et al. 2013). Fluorescence emission is expressed in relative fluorescence units (RFU).

High-temperature catalytic oxidation (HTCO) in a Shimadzu Total Organic Carbon Analyzer (TOC-L, Shimadzu Corp., Kyoto, Japan) was used to estimate TOC in seawater (Calleja et al. 2019). One hundred microliters of replicate samples were injected into the pre-heated (680 °C) combustion tube. A five-point potassium hydrogen phthalate calibration curve was used for instrument standardization. The Consensus Reference material of deep-sea water (42–45 μmol C L^−1^) and low carbon water (1–2 μmol C L^−1^) was used to monitor the accuracy of the measurements allowing a resolution of 1.4 μmol L^−1^ (Calleja et al. 2019).

The concentrations and isotopic composition of CH_4_ and CO_2_ were measured by cavity ring-down spectroscopy (CM-CRDS G2201-I, Picarro Inc., Santa Clara, CA, USA) by the closed water circuit technique (Sea et al. 2018). Briefly, the water samples were recirculated in an enclosed water circuit through a membrane equilibrator (Liqui-Cel MiniModule, 3M, Minnesota, USA) to establish an equilibration of gases in dissolution. The air from the enclosed air circuit was subsequently passed through a desiccant column (calcium sulfate, WA Hammond Drierite Co., LTD, Ohio, USA) and flowed into the CRDS system to record the isotopic carbon composition of CO_2_ and CH_4_ in the sample (Burkholz et al. 2020). The analytical precision of δ^13^C*–*CH_4_ and δ^13^C*–*CO_2_ measurements was ±1.5‰ and ±0.2‰, respectively. We used an industrial air mixture (750 ppm CO_2_ and 9.7 ppm CH_4_; Abdullah Hashim Industrial Gases & Equipment Co. Ltd., Jeddah, Saudi Arabia) as a standard.

We estimated 18 PAHs (16 EPA PAH, 1-methylnapthalene, and 2-methylnapthalene) in seawater by solid-phase extraction (SPE) using an EnvirElut-PAH cartridge (1 gm/6 mL, Agilent, Santa Clara, CA), followed by gas chromatography tandem triple quadrupole mass spectrometry (GC-MS/MS, Agilent 7890 GC 7010B/MS). Briefly, 25 mL of the preserved water samples were cleaned up by SPE using the EnvirElut-PAH column, pre-conditioned with methanol and Milli-Q water. PAHs were eluted with cyclohexane, and 1 μL of the extract was injected into a DB-EU PAH 20 m. 0.18 mm, 0.14-μm film thickness column. PAHs were quantified with a practical quantitation limit (PQL) of 0.04 μg L^−1^, using an external calibration curve of PAHs ranging from 1 to 100 ng mL^−1^ in dichloromethane with internal PAH standard at 100 ng mL^−1^ and QC/QA according to US-EPA Method 8270 D. The transitions used for PAH determination are shown in the Supplementary materials Table S1.

Concentrations of major and trace elements in seawater were directly measured using inductively coupled plasma-optical emission spectroscopy (ICP-OES) bearing a synchronous vertical dual view configuration and a wavelength window between 167 and 785 nm (model: 5110 VDV, Agilent Technologies, USA), following the modified US-EPA Method 200.7. The sample introduction system consisted of a SeaSpray nebulizer, a double-pass glass cyclonic spray chamber, and a standard 1.8-mm ID injector torch. All measurements were performed in axial plasma viewing mode at a pump speed of 10 rpm. The instrument was standardized by measuring the quality control standards (QCS) and acid blank spiked with element standard solutions (Simoes et al. 2020) within the recovery rates of 95–105%, while the measured element concentrations of the standard reference materials were in good consistency with the certificated values (77.51–113.02%).

### Estimation of bacterial abundance

Enumeration of bacteria in the seawater was carried out using a BD FACSCanto II flow cytometer (BD Biosciences, Eysins, Switzerland). Bacteria cells were stained at room temperature with SYBR Green (1% v/v) and dark-incubated for 15 min. For absolute counting of target cells, a known number of fluorescent beads (1 μm diameter, 1 × 10^6^ beads/mL) were added, and the samples were acquired at a low flow speed for 60 s. High nucleic acid (HNA) and low nucleic acid (LNA) bacteria populations were distinguished based on their side scatter and green fluorescence signal (SSC-H vs. FITC-A 4-log decade dot plots). The abundances were calculated based on the flow rate, compensating for the dilution effect of fixative, stain, and beads solutions. All data were batch-processed using FCS Express 6 RUO software (De Novo Software, Glendale, CA).

### Biochemical analyses of zooplankton biomass

For elemental analysis (Cai et al. 2022), 200 mg of freeze-dried (FreeZone 18 Liter Console, Labconco, USA) sample was digested in a mixture of nitric acid (trace metal analytical grade; TraceSELECT® Ultra, Sigma-Aldrich) and hydrogen peroxide (5:1 v/v) using a single reaction chamber microwave digestion system (1500 W, model ultraWAVE, Milestone Technologies, USA), applying successive steps of heating at 130 °C for 10 min and 240 °C for 25 min at 100 bar, 98.69 standard atmospheric pressure. The digested biomass was then diluted to 25 mL by Milli-Q distilled water, and heavy metal concentrations were analyzed using inductively coupled ICP-OES as described in the “Spectroscopic and chemical analyses of oil signals in seawater” section. To ensure the validation of data as well as the accuracy and precision of the analytical methods, 3 replicates of mussel tissue standard reference material (SRM 2976, National Institute of Standards and Technology, USA) were also digested and measured along with the zooplankton samples. The limit of detection (LOD) was 0.019 mg/kg.

The TOC and total nitrogen (TN) in zooplankton samples were analyzed using the CHNS Organic Elemental Analyzer (Flash 2000, Thermo Fisher Scientific, USA). Briefly, 10 mg freeze-dried samples were digested in 10 μL 3 mol/L HCl (Fisher Chemical, USA) in silver capsules (10 mm×10 mm, OEA labs, UK). The capsules with biomass were then heated at 60 °C for 10 min to remove all inorganic carbon contents. The samples were acidified with HCL until no further bubbles appeared in the silver capsules. Excessive HCL was allowed to evaporate completely at room temperature overnight. The silver capsules were subsequently wrapped in tin capsules (10 mm×10 mm, OEA Labs, UK) and introduced to the CHNS analyzer. Standard curves for TOC and TN were obtained by measuring 2–10 mg aspartic acid standards (C_4_H_7_NO_4_, Thermo Fisher Scientific, USA) wrapped in tin capsules.

PAHs in the zooplankton biomass were extracted by pressurized liquid extraction (PLE) with dichloromethane as a solvent in a Dionex^TM^ ASE^TM^ Accelerated Solvent Extractor (Thermo Fisher Scientific, Waltham, MA, USA) as described in Supplementary Method S3. Extracts were concentrated using a Genevac Rocket Evaporator (SP Industries, Warminster, PA, USA) and cleaned up by gel permeation chromatography (GPC) using a Robotic System FREESTYLE (LCTech GmbH, Obertaukirchen, Germany). Concentrations of 18 PAHs (16 EPA PAH, 1-methylnapthalene, and 2-methylnapthalene) in the clean extract were estimated by gas chromatography/tandem triple quadrupole mass spectrometry (GC-MS/MS, Agilent 7890 GC 7010B/MS) according to US-EPA Method 8270 D. One microliter of the extract was injected into a DB-EU PAH 20 m. 0.18 mm, 0.14-μm film thickness column. We used an external calibration curve for PAHs ranging from 1 to 100 ng mL^−1^ in dichloromethane with an internal PAH standard at 100 ng mL^−1^ and a practical quantitation limit (PQL) of 0.01 μg L^−1^.

### Statistical analysis

All statistical analyses were conducted using JMP software (JMP® Pro version 14.1, SAS Institute, USA). Due to the small sample size, non-parametric Wilcoxon/Kruskal–Wallis tests (rank sums) were performed, followed by a post-hoc Steel–Dwass method to test for significant differences among samples from different sampling stations. A statistically significant level of 0.05 was used.

## Results

### Optical properties of seawater in the spill vicinity

**Fig. 2:**
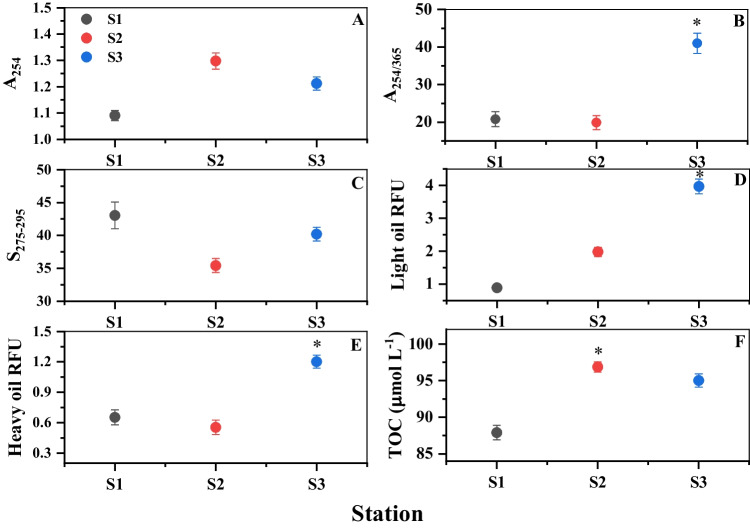
Shift in optical properties and organic carbon level of the Red Sea surface water in the oil spill vicinity. **A** CDOM absorbance coefficient a_254_; **B** absorption coefficient ratio at 254/365; **C** spectral slope of CDOM in the 275–295 nm range; **D** light oil fluorescence emission; **E** heavy oil fluorescence emission; **F** TOC. Values are given as mean ± SE. Asterisks indicate a significant difference from S1 (Wilcoxon *p*< 0.05)

The oil spill significantly altered the optical properties of dissolved organic matter (DOM) components in the affected seawater (Fig. 2). CDOM absorbance coefficient (a_254_) in the vicinity of the oil spill was 10–20% higher (Wilcoxon/Kruskal–Wallis test: *χ*^2^  =  7.20, df  =  2, *p*  =  0.027) than 1.09 m^−1^ recorded at S1 (Fig. 2A). The absorption coefficient ratio at 254/365 nm was higher in the spill vicinity than that in the furthest area at S1 (Fig. 2B), but the difference was not significant (Wilcoxon/Kruskal–Wallis test: *χ*^2^  =  5.42, df  =  2, *p*  =  0.067). The spectral slope of CDOM in the 275–295 range was significantly higher (Wilcoxon/Kruskal–Wallis test: *χ*^2^  =  7.2, df  =  2, *p*  =  0.028) at S1 (Fig. 2C).

The fluorescence emission of the seawater indicated the presence of light and heavy oil components. The light oil fluorescence emission increased significantly toward the spill site (Wilcoxon/Kruskal–Wallis test: *χ*^2^  =  7.2, df  =  2, *p*  =  0.028). The RFU value at S3 increased over fourfold than that in the unaffected region (Fig. 2D). Similarly, the heavy oil fluorescence (Fig. 2E) recorded an 84% increase from S1 at the station close to the spill area (Wilcoxon/Kruskal–Wallis test: *χ*^2^  =  5.95, df  =  2, *p*  =  0.048).

### Chemical analyses of seawater in the spill vicinity

Oil spill significantly increased dissolved TOC levels in the Red Sea waters (Fig. 2F). Mean TOC was 4–8% higher in the spill-affected waters than the ambient levels (Wilcoxon/Kruskal–Wallis test: *χ*^2^ =  6.49, df  =  2, *p*  =  0.039).

The mineral composition of seawater in the spill vicinity remained unaffected after the oil spill (Supplementary materials Fig. S3). According to the Wilcoxon/Kruskal–Wallis test, the concentrations of all major minerals in the spill proximity were statistically similar to those in the unaffected region (*p* > 0.05). The order of concentration of major elements in the Red Sea surface water was Na > Mg > S > Ca > K. All other trace elements including heavy metals were recorded below their detection limits.

**Fig. 3:**
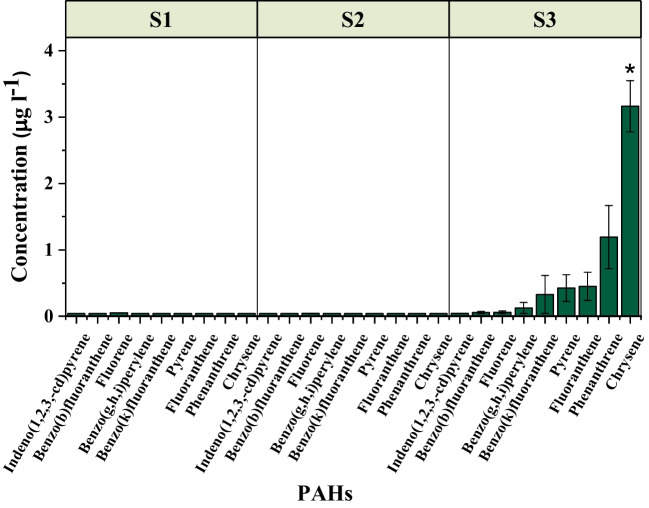
Increase in the surface water PAH levels in the Red Sea after the oil spill. The values represent mean ± SE, and an asterisk indicates a significant difference from S1 (Wilcoxon *p* < 0.05). The detection limit was 0.04 μg L^−1^

Out of 18 PAHs analyzed, we detected 9 in the spill-affected Red Sea above the detection limit (0.04 μg L^−1^). No PAHs were detected at stations S1 and S2 except for traces of the three-ringed fluorene (Fig. 3). However, significantly higher levels of 3–4 ringed PAHs (chrysene, phenanthrene, fluoranthene, and pyrene) along with traces of high molecular weight PAHs (benzo(b)fluoranthene, benzo(g,h,i)perylene, and indeno(1,2,3-cd)pyrene) were detected at S3, which was closer to the original spill site.

The isotopic composition of DOC (δ^13^C) after the spill showed a shift toward the δ^13^C value of the crude oil, from the baseline concentrations observed prior to the spill in the Red Sea (Supplementary materials Table S2). The baseline δ^13^C values of Red Sea water were slightly positive at 0.88‰ which was significantly depleted at spill-affected sites. The δ^13^C–CO_2_ was lowest (−8.42‰) at S2. However, the δ^13^C–CH_4_ was comparable across the two spill-affected stations.

### Microorganisms and zooplankton

**Fig. 4:**
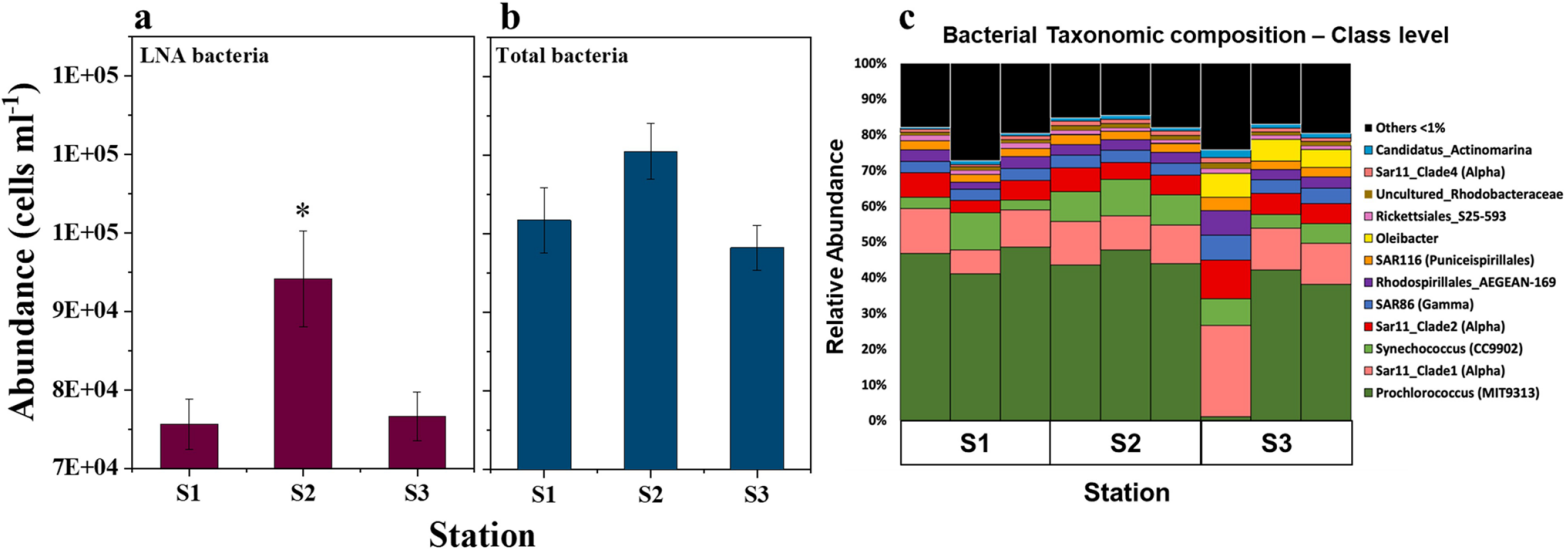
Changes in the abundances of LNA bacteria and total bacteria in the Red Sea after exposure to the oil spill (**a**) and the corresponding 16S rRNA gene amplicon sequencing taxonomy at the class level (**b**). Values ((a) and (b)) represent mean ± SE, and an asterisk indicates a significant difference at Wilcoxon *p* < 0.05

We found that the oil spill caused apparent imbalances in the bacterial and picophytoplankton communities in the Red Sea. The abundance of LNA bacteria was 25% higher at station 2 (Wilcoxon/Kruskal–Wallis test: *χ*^2^  =  6.49, df  =  2, *p*  =  0.039), in accordance with higher A_254_ and TOC values than those in the unaffected region (Fig. 4(a)). However, the HNA counterpart remained at statistically similar levels across the stations studied. Although the mean total bacterial abundance was higher at station 2 (Fig. 4(b)), the Wilcoxon/Kruskal–Wallis test did not show any significant difference among stations (*χ*^2^  =  4.62, df  =  2, *p*  =  0.099).

The bacterial community analyzed by 16S rRNA gene sequencing across three sampling stations (Fig. 4(c)) was mainly composed of cyanobacteria belonging to genera *Prochlorococcus* (38–48% of relative abundance) and *Synechococcus* (3–10%); Alphaproteobacteria, including different clades of Sar11 (7–25%); and Rhodospirillales (1–3%). At station 3, a bloom of *Oleibacter* (Gammaproteobacteria) was detected in the community with a relative abundance between 5 and 6.7%. In one replicate of station 3, the abundance of *Prochlorococcus* was drastically lower (~1%) than that at the other stations. Despite this difference, the overall bacterial composition was maintained in this sample, and it confirmed the presence of *Oleibacter* only in the close vicinity of the spill. In addition, the oil spill resulted in an increase in the alpha diversity of the communities at station S3 (Supplementary materials Fig. S4).

**Fig. 5:**
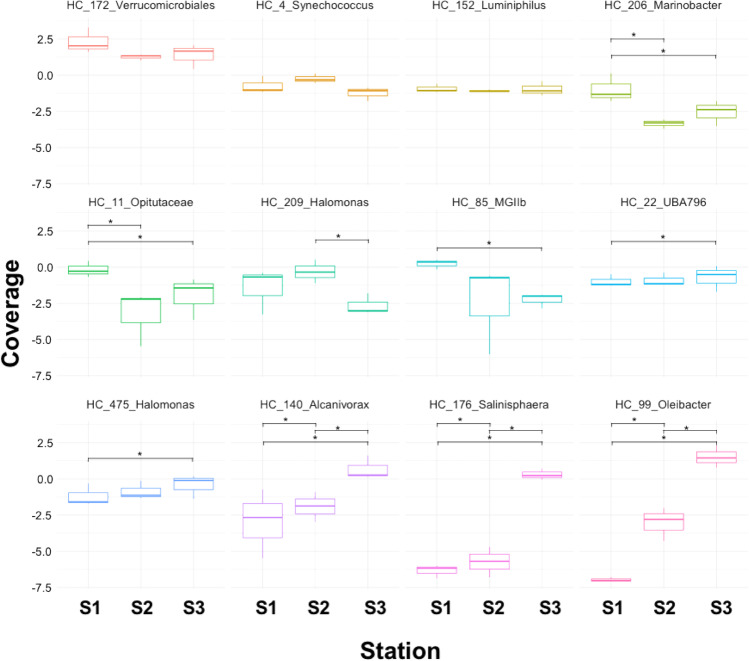
Boxplot of significant differential enriched MAGs present in the Red Sea after the oil spill. Data from metagenomic MAG assembly. * indicates a significant difference at *p* < 0.05

From all the samples, we reconstructed 54 high-quality MAGs (Konstantinidis et al. 2017) by metagenomic sequencing and analysis. Forty-six MAGs were assigned to bacteria and 8 to Archaea (Supplementary materials Fig. S5). Among such MAGs, a significant increase in the reads of bacteria capable of alkane degradation such as *Alcanivorax* (DESeq2 log2 fold change = −4.04; p_adj_ = 1.7E−02), *Salinisphaera* (DESeq2 log2 fold change = −10.22; p_adj_ = 2.4E−13), and PAH-degrading bacteria belonging to the genera *Oleibacter* (DESeq2 log2 fold change = −13.58; p_adj_ = 1.3E−26) were enriched at station S3. On the contrary, *Halomonas* were significantly decreased at station 3 (DESeq2 log2 fold change = 3.49; p_adj_ = 1.32E−03). The presence of widespread surface seawater bacteria, which generally dominate unperturbed communities such as *Synechococcus*, was not affected by the oil spill (Fig. 5 and Supplementary materials Table S3). The analyses of the MAGs significantly enriched, especially in station 3, confirmed that all possessed genes were involved in alkane degradation. Furthermore, the *Alcanivorax* strain contains genes involved in the degradation of naphthalene and phenanthrene. We also noted that 8 other MAGs (not significantly abundant) possess some genes involved in PAH degradation (i.e., phthalate, benzene, naphthalene, and phenanthrene).

**Fig. 6:**
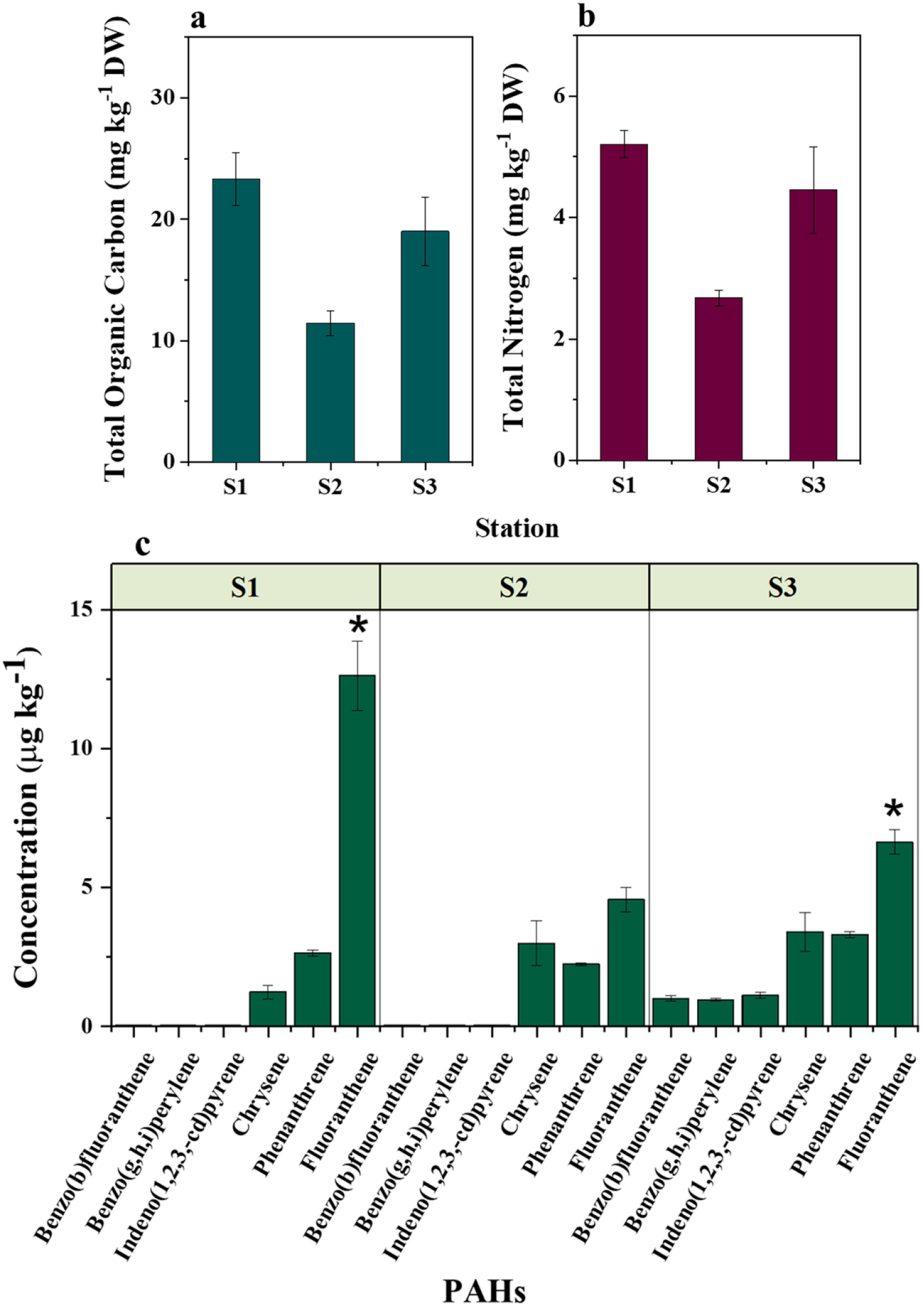
Variation in TOC (**a**), total nitrogen (**b**), and PAH (**c**) levels in the zooplankton biomass collected in the Red Sea after exposure to the oil spill. Asterisks indicate a significant difference (Wilcoxon *p*< 0.05)

One week after the spill, we found significantly lower levels of TOC (Wilcoxon/Kruskal–Wallis test: *χ*^2^  =  5.95, df  =  2, *p*  =  0.044) and TN (Wilcoxon/Kruskal–Wallis test: *χ*^2^  =  6.95, df  =  2, *p*  =  0.037) in zooplankton biomass collected in the stations at the vicinity of the spill, compared to the unaffected region (Fig. 6a, b). TOC and TN in the zooplankton biomass at S2 were respectively reduced up to 51% and 49% than those found at S1.

Out of the nine PAHs detected in the seawater, six PAHs were found in zooplankton collected at the spill premises in the Red Sea (Fig. 6c), and the major PAHs were in the order of fluoranthene > phenanthrene > chrysene. High molecular weight PAHs such as benzo(b)fluoranthene, benzo(g,h,i)perylene, and indeno(1,2,3-cd)pyrene were detected only at the close premise of the spill. Moreover, a significant correlation was observed between PAH concentrations in seawater and the zooplankton biomass samples (*r*^*2*^ = 0.67; *p* = 0.02).

The mean concentration of major minerals in the zooplankton biomass in the unaffected region of the Red Sea was in the order, Na > Ca > S > K > Mg > P (Table 1). Concentrations of S in zooplankton biomass did not vary significantly across sampling stations (Wilcoxon/Kruskal–Wallis test: *χ*^2^  =  1.42, df  =  2, *p*  =  0.491). However, significant increases in Mg (Wilcoxon/Kruskal–Wallis test: *χ*^2^  =  3.86, df  =  2, *p*  =  0.049), K (Wilcoxon/Kruskal–Wallis test: *χ*^2^  =  3.67, df  =  2, *p*  =  0.047), Na (Wilcoxon/Kruskal–Wallis test: *χ*^2^  =  7.20, df  =  2, *p*  =  0.027), and Ca (Wilcoxon/Kruskal–Wallis test: *χ*^2^  =  6.94, df  =  2, *p*  =  0.031) were observed at S2. At S3, all the major minerals were significantly depleted than those in the unaffected sample, except for Ca.

**Table 1:** Elemental concentrations (mean ± SE) in the Red Sea zooplankton sampled at stations in the vicinity of the oil spill

Element	Concentration (mg kg^−1^ DW)		
	Station 1	Station 2	Station 3
Aluminum (Al)	921 ± 109	465 ± 121	1936 ± 866
Arsenic (As)	21.2±3.2	17.5±4.2	25.4 ± 1.2
Cadmium (Cd)	3.5 ± 0.5	1.4 ± 0.2	2 ± 0.4
Calcium (Ca)	40393 ± 1593	92025 ± 7633	126909 ± 2601
Chromium (Cr)	41.4 ± 17.9	6.26 ± 1.7	16 ± 4.9
Copper (Cu)	31.4 ± 7.4	210 ± 63.2	765 ± 349
Iron (Fe)	5097 ± 2510	2778 ± 801	1974 ± 430
Magnesium (Mg)	9026 ± 463	14406 ± 197	7623 ± 224
Manganese (Mn)	51.0 ± 9.1	24.5 ± 3.5	37 ± 5.1
Nickel (Ni)	22.2 ± 3.8	4.8 ± 0.7	15.4 ± 3.8
Phosphorus (P)	4557 ± 179	2575 ± 68	3959 ± 440
Potassium (P)	11698 ± 1167	18039 ± 1880	8072 ± 367
Sodium (Na)	70160 ± 2747	102749 ± 5530	40719 ± 3079
Strontium (Sr)	12279 ± 140	5770 ± 99	9145 ± 500
Sulfur (S)	12297 ± 507	12039 ± 537	11527 ± 669
Zinc (Zn)	3663 ± 2326	657 ± 50	531 ± 80

We detected clear fluctuations in trace element concentrations in zooplankton biomass after the oil spill (Table 1). The toxic heavy metal Cu sharply increased from 31.4 ± 7.4 up to 765 ± 349 mg kg^−1^ DW in samples collected at the spill vicinity (Wilcoxon/Kruskal–Wallis test: *χ*^2^  =  7.3, df  =  2, *p*  =  0.029). However, Wilcoxon/Kruskal–Wallis test revealed that levels of Sr (*χ*^2^  =  7.2, df  =  2, *p*  =  0.027), Zn (*χ*^2^  =  6.1, df  =  2, *p*  =  0.047), and Cr (*χ*^2^  =  7.2, df  =  2, *p*  =  0.027) were significantly lower in the spill-affected samples. The rest of the trace elements such as Ni, Cd, As, Al, Mn, and Fe did not show significant variation across the collected samples (Wilcoxon/Kruskal–Wallis test *p* > 0.05).

## Discussion

The Red Sea is vulnerable to oil pollution from accidental spills along its main shipping lanes. The atmospheric and oceanographic features of the Red Sea, along with its relatively narrow size, determine the spread and transport of spilled oil across the basin (Mittal et al. 2021), resulting in localized impacts on coastal areas with pristine ecosystems, livelihoods, fisheries, and desalination facilities (Kleinhaus et al. 2020a). However, despite a high susceptibility to oil pollution risks (Kleinhaus et al. 2020b; Mittal et al. 2021), a comprehensive understanding of oil spill impacts in the Red Sea environment is lacking. In this study, we report significant changes in seawater characteristics and exposure of planktonic organisms to bioavailable oil contaminants in the close vicinity of the 2019 oil spill in the central Red Sea.

Crude oil is a heterogeneous mixture of hydrocarbon-type organic molecules, with high structural complexity, comprised of saturated hydrocarbons, aromatics, resins, and asphaltenes (Abbriano et al. 2011; Brussaard et al. 2016). We found that the water-soluble fraction of the spilled oil quickly enters seawater leading to an elevation of dissolved carbon and significant changes in the optical properties of seawater, as previously shown in the Gulf of Mexico following the Deepwater Horizon oil spill (D’Sa et al. 2016; Zhou & Guo 2012; Ziervogel et al. 2012). A significant increase in bulk CDOM signals in the close vicinity of the spill (Fig. 2A, B, C) indicates degradation and dissolution of the water-soluble fraction (WSF) of crude oil in the earlier days after the event, which contributed to TOC amendment in the seawater.

The isotopic dilution of δ^13^C in the spill-affected region confirms the degradation of a very negative source, the spilled oil. Being a natural product depleted in ^13^C, crude oil has a strongly negative δ^13^C, normally within a range of −32 to −24 (Barrie et al. 2016; Medina-Bellver et al. 2005; Wang et al. 2013); therefore, the dissolution of carbon from the crude oil and subsequent degradation to ^13^C-depleted CO_2_ result in further decline in the δ^13^C of the dissolved carbon. The background δ^13^C–CO_2_ value of the unaffected Red Sea water was close to 1 (Supplementary materials Table S1) which corresponds to those reported in the Atlantic and various other oceanic regions (Kroopnick 1985; Medina-Bellver et al. 2005). Thus, the isotopic data confirm the prevailing biological activities in the spill-affected region that rapidly degrade oil compounds in the DIC.

In the Red Sea, the concentration of CDOM is relatively very low due to the arid climatic conditions and lack of riverine inputs (Overmans & Agustí 2019). The high CDOM fluorescence at S2 and S3 evidences the freshly spilled oil as an additional source material and the associated microbial and photochemical oil degradation processes. Hence, CDOM fluorescence analysis offers rapid spill detection in the Red Sea, particularly in oligotrophic offshore waters where the influence of coastal CDOM sources is at a minimum. In addition, the observed strong relationship between rises in TOC and CDOM in the spill vicinity (S2) reveals the incorporation of optically active hydrocarbons from the spilled oil. The spatial pattern of CDOM absorbance in the spill vicinity demonstrates the difference in CDOM composition and quantitative status of oil degradation processes across the affected area. The CDOM represents an important reactive component of DOM (Bowen et al. 2017), the amount, functionality, and sources of which were significantly transformed in the oil spill-affected region of the Red Sea. Moreover, the relatively lower S_275–295_ at S2 (Fig. 2C) indicates an increase in optical activity and aromaticity degree typical of PAH compounds (Gonnelli et al. 2016).

PAHs represent many of the most toxic components of crude oil that bioaccumulate and biotransfer to marine invertebrates (Wassenaar & Verbruggen 2021). Oil spill sites are prominent sources of PAHs with a clear spatial trend centered at the spill site to the surrounding seawater (Liu et al. 2013; Liu et al. 2016). Given the high susceptibility of the Red Sea basin to oil contamination, due to intermittent accidental spills, in particular, elevated coastal PAH levels (> 10 μg L^−1^) are likely at certain sites in the Red Sea, dominated by four-ringed, high molecular weight PAHs (Al-Mur 2019; El-Naggar et al. 2021). We recorded traces of 3-ringed (phenanthrene), 4-ringed (pyrene, fluoranthene, chrysene), and 5-ringed (benzo(k)fluoranthene) PAHs in the close vicinity of the spill site. This is similar to a previous study reporting that the low to medium molecular weight PAHs of the oil are susceptible to rapid weathering and dissolution into the dissolved phase at oil spill sites (Sammarco et al. 2013). PAH levels in oil spill-affected marine environments are highly dynamic, both spatially and temporally, due to variations in solubilization, photodegradation, weathering, and biodegradation of individual compounds (Boehm et al. 2016). Photooxidation of PAHs is a relevant immediate fate of oil-derived PAHs in surface seawater where the transformation of saturated and aromatic fractions to more polar, oxygen-containing species could be observed (Radović et al. 2014).

The aromatic fraction of crude oil is characterized by strong fluorescence emission in the UV region, as shown by the drastic increase in the fluorescence signatures of the light oil in the affected area (Fig. 2D). Such fluorescence increase corresponds to the detection of 9 PAHs, including persistent high molecular weight compounds, at the close vicinity of the spill in significantly higher concentrations than the background levels. Moreover, a high heavy oil fluorescence indicates the presence of polar components, a sign of crude oil biodegradation (Head et al. 2006). Aromatic hydrocarbons and polar fractions in seawater are a major long-term threat to the marine environment due to their long-term persistence and toxicity (Kim et al. 2019). The spatial and temporal distribution of these toxic components in the basin will depend on the mode of response measures, physical factors such as oceanic currents, and the rate of oil degradation. The weathering/degradation of spilled oil is affected by climatic conditions, nutrient availability, and microbial dynamics in the surface seawater of the affected area (Passow & Overton 2021). For instance, if an oil spill in the Red Sea occurs in winter, the strong winter currents can bring oil dispersion from south to north resulting in extensive damage to coastal ecosystems over a larger area (Kleinhaus et al. 2020b), exacerbating the prevailing hydrocarbon contamination of pristine coastal habitats including mangrove swamps and coastal lagoons (El-Maradny et al. 2021). These impacts will last long term, in particular, those affecting the fluorescence pool of the DOM (D’Sa et al. 2016).

Tracking the impacts of oil spills on marine microbiota is a significant aspect to comprehend the ecological consequences of the catastrophe. Microbial communities show a rapid response to oil contamination due to their relatively short generation times (Gemmell et al. 2018). Our analysis after 6 days of the spill event revealed no significant shifts in total heterotrophic bacterial abundance. The surface microbial community in the Red Sea was dominated by *Synechococcus* and *Prochlorococcus* and different strains of the Alphaproteobacteria SAR11 clade (Coello-Camba & Agustí 2021; Thompson et al. 2013; Thompson et al. 2019). The oil spill affected the community composition, and after 6 days from the spill, we observed an enrichment of n-alkane degrading bacteria such as *Alcanivorax* (Kostka et al. 2011), *Salinisphaera* (Wang et al. 2010), and *Oleibacter* (Lofthus et al. 2018; Teramoto et al. 2011). Furthermore, at S3, an increase in the community diversity occurred, presumably as a consequence of the selection imposed by the alteration of the nutrients in the seawater and the effects of the oil hydrocarbons released with the spill that have both favorable (source of substrates for growth) and detrimental (toxicity) effects on different microorganisms of the community. This may subsequently promote the growth of specific hydrocarbon-degrading organisms and a decline of the species that are sensitive to oil hydrocarbon contamination. The natural seepage of crude oil and gases from sedimentary reservoirs on the Red Sea floor contributes to submarine hydrocarbon levels (Bourtsoukidis et al. 2020), and the native picophytoplankton populations in the Red Sea are comparatively more resilient to their adverse impacts (Kottuparambil & Agusti 2018). This is consistent with an increase in the communities of microorganisms adapted to exploit the environmental conditions that the oil spill generates. Interestingly we observed a growth of different microbial species involved in alkane degradation, only at  the station close to the spill location where we observed a higher concentration of hydrocarbons. The enrichment of different species involved in hydrocarbon degradation could be explained by the resilience of the Red Sea microbial community that presents a redundancy in the oil degradation capacity that is quickly triggered by the pollution event as shown by the presence of more than 10 MAGs that possess genes involved either in alkane or PAH degradation.

An opposite trend was observed for marine zooplankton. In general, marine zooplankton communities respond rapidly to exposure to oil by an abrupt decline in diversity and abundance (Brussaard et al. 2016), along with mortality and sublethal effects over time (Daly et al. 2021). However, microzooplankton communities can help extend the residence time of toxic oil components in the euphotic zone through accumulation into their biomass. The minute crude oil droplets formed by physical or chemical processes during an oil spill are usually in the size range of food particles for several zooplankton species; therefore, their ingestion by these organisms (Campelo et al. 2021) represents an important intake pathway of petroleum contaminants into the marine food web (Almeda et al. 2014). We found traces of six PAHs in the zooplankton biomass collected at the Red Sea spill area, including three high molecular weight PAHs (benzo[b]fluoranthene, benzo[g, h, i]perylene, and indeno[1, 2, 3-cd]pyrene). The lipid solubility of PAHs increases as their molecular weight increases, leading to accumulation in the lipid tissues of organisms (Juhasz & Naidu 2000). Higher concentrations of individual PAHs in the Red Sea zooplankton at station 3 (Fig. 6c) correlated with their higher octanol-water coefficient (log K_ow_ ranged from 4.57 to 7.10), in agreement with a previous study where the selective accumulation of fluoranthene, pyrene, chrysene, and benzo[b]fluoranthene was observed in zooplankton exposed to crude oil emulsion (Almeda et al. 2013). Accumulation of high molecular weight, lipophilic PAHs in primary consumers brings threat to higher trophic levels such as fish due to the high persistence, biomagnification potential, and carcinogenic and mutagenic properties of these compounds (Sachaniya et al. 2019). However, the extent of bioaccumulation of PAHs in marine organisms depends on the ability of the species to metabolize, biotransform, and depurate the hydrocarbons by excretion through fecal pellets (Almeda et al. 2013) or egg production (Berrojalbiz et al. 2009). For example, the degradation rates of low molecular weight and less hydrophobic PAHs are comparatively higher in zooplankton due to high metabolic intake rates (Berrojalbiz et al. 2009). Moreover, high solar UV irradiation prevalent in the Red Sea (Overmans & Agustí 2019) can have additional consequences for bioavailability and transformation of PAHs in the marine environment.

Marine oil spills result in elevated heavy metal levels such as copper in surface seawaters (Santos-Echeandía et al. 2008) and marine organisms (Bu-Olayan & Subrahmanyam 1997). Heavy metal accumulation in zooplankton biomass in the Red Sea has been significantly impacted by the oil-related industrial effluents from coastal urban areas (Cai et al. 2022). For instance, Cu levels in the Red Sea zooplankton biomass ranged between 7 and 53 mg kg^−1^ DW where the highest concentrations were detected in the northern region close to a major petrochemical industrial area (Cai et al. 2022). Oil spills contribute to increased bioaccumulation of toxic metals in marine biota (Bu-Olayan & Subrahmanyam 1997), and the sharp increase in Cu concentration in zooplankton (Table 1) likely indicates acute toxic metal contamination in key marine organisms at the oil spill proximity in the Red Sea. Zooplankton are typically short-lived organisms that are rapidly consumed by higher trophic–level animals leading to the subsequent transfer of the bioaccumulated toxic compounds to the marine food web (Buskey et al. 2016). Thus, contamination of the lower trophic organisms in the spill-affected Red Sea can subsequently impact and deteriorate the quality of fisheries and seafood and evoke human health risks due to the large-scale harvest and consumption in the area (Said 2007; Shellem et al. 2021).

Accidental spills in the ocean can immediately contaminate surface water down to 8–500 m below the visible surface slick, depending on the intensity of wind and currents (Brussaard et al. 2016). However, sampling after oil spill events in the ocean is complex and challenging due to limited resources, unforeseen dynamics of the spilled oil, and a paucity of information. For example, in our study, we were unable to collect the source oil to identify the specific type of hydrocarbon released to the Red Sea or to identify any other potential source of hydrocarbon pollution within the spill area. This limited further oil forensic analyses that could have provided more characteristic details of the contamination.

Additionally, our analysis neglected the potential for natural weathering and dispersion of spilled oil on the sea surface by winds and currents over time. The extent of the consequences of this underestimation depends on the type of source oil and climatic conditions, such as temperature and sunlight during the event. For example, the evaporation of spilled oil is a critical aspect that regulates the amount of oil on the sea surface. Furthermore, we omitted important ecological impacts of oil exposure in the photic zone, such as the decline in the plankton population that can indirectly affect the long-term structure and function of the marine food web.

Although there are limitations, our data suggest that oil spills cause significant responses in fundamental trophic groups in the ocean. Our study provides valuable baseline information for policymakers, scientists, and conservationists working to monitor the impacts of oil spills on the marine environment. Early detection of oil carbon in seawater and plankton, as well as the enrichment of specific bacterial genera in the seawater microbiome, can provide significant insights into the fate and long-term effects of oil spills. However, the immediate impacts of sudden oil releases may not be documented due to typical delays in sampling and investigation. Addressing this important aspect of open ocean spills requires better real-time oil contamination monitoring systems, which could potentially improve immediate mitigation action plans for marine regions such as the Red Sea, where intermittent oil spill accidents of varying magnitudes are expected.

An important future direction for oil spill research in the Red Sea is to pay more attention to small- and medium-sized spill accidents since their frequent occurrence in the region makes them as hazardous as major spills. A more comprehensive approach would be to include satellite imagery and other remote sensing technologies to identify spills that may remain undetected. Moreover, the responsibility and jurisdiction mechanisms of the coastal countries of the Red Sea should be improved. This will strengthen surveillance and management in this region and minimize accidents in the future. In addition, there needs to be a greater focus on the long-term effects of oil spills on the marine ecosystem. This includes studying the impact on fish populations, coral reefs, and other important marine life.

## Conclusions

Oil contamination caused by accidental/industrial spillages is among the major persistent regional concerns in the Red Sea (Kleinhaus et al. 2020b). However, the early impacts of oil spills are often poorly documented due to the typical delay in prompt on-site sampling due to logistical issues (Brussaard et al. 2016; Burton Jr et al. 2021). We detected significant signals of the 2019 Red Sea oil spill in seawater within a one-week period from the spill. Our observations suggest that even a short-lived spill, such as the one that lasted for a few days in this case study, can cause significant impacts on various segments of the marine environment. During the early days of the spill, the dissolution of fresh crude oil resulted in elevated DOC levels with high optical reactivity, exhibiting clear signals of dissolved fluorophores in the surface seawater. We demonstrated that fluorescence-based optical methods provide a rapid, sensitive, and efficient way to track the in vivo impacts of crude oil in seawater. The instant amendment of organic matter in the seawater fuels complex shifts in planktonic composition and structure in the euphotic zone. Although oil spills can temporarily boost phytoplankton growth and primary/secondary production in the affected areas, the rapid entry of less soluble and potentially toxic petroleum hydrocarbons into the marine biosphere has ecological, economical, and human health consequences. Particularly, the ingestion of persistent oil-derived contaminants including PAHs and heavy metals by epipelagic zooplankton represents their primary pathway of entry into the food chain. This study emphasizes that immediate survey and investigation must be integrated as an obligatory part of the oil spill response actions for pristine marine hotspots like the Red Sea.

## Supplementary information

ESM 1 (DOCX 289 KB)

## Data Availability

All data are provided in the manuscript.
