# Supplementary material for: Tracking the early signals of crude oil in seawater and plankton after a major oil spill in the Red Sea
Source: Environ Sci Pollut Res Int. 2023 May 3;30(26):69150–64. doi: 10.1007/s11356-023-27111-0 (PMC10212844; doi:10.1007/s11356-023-27111-0)
Supplement: Supplementary file 1 — (DOCX 289 KB) [file 11356_2023_27111_MOESM1_ESM.docx]

**Supplementary Materials**

**Tracking the early signals of crude oil in seawater and plankton after a major oil spill in the Red Sea**

**Sreejith Kottuparambil^1^*, Ananya Ashok^1^, Alan Barozzi^1^, Grégoire Michoud^1^, Chunzhi Cai^1^, Daniele Daffonchio^1^, Carlos M. Duarte^1, 2^, Susana Agusti^1^**

^1^Red Sea Research Center (RSRC), King Abdullah University of Science and Technology (KAUST), Thuwal, Saudi Arabia.

^2^ Computational Bioscience Research Center (CBRC), King Abdullah University of Science and Technology, Thuwal, Saudi Arabia.

*** Correspondence:**Sreejith Kottuparambil
[sreejith.kottuparambil@kaust.edu.sa](mailto:sreejith.kottuparambil@kaust.edu.sa)

**List of Methods**

Method S1. DNA extraction and metagenomic library preparation

Method S2. Genome analysis

Method S3. Estimation of PAHs in zooplankton biomass

**List of Tables**

Supplementary Table S1. Retention time (RT), internal standards (ISTD), transitions and collision energies (CE) used for the determination of PAHs.

Supplementary Table S2. CO_2_, CH_4_, and δ^13^C levels in seawater at the spill-affected Red Sea.

Supplementary Table S3. Variation in the MAGs in the oil spill site in the Red Sea.

**List of Figures**

Supplementary Figure S1. 16S rRNA gene amplicon sequencing number of reads per samples.

Supplementary Figure S2. Rarefaction curve for samples at the oil spill site in the Red Sea.

Supplementary Figure S3. Variation in major mineral levels in surface water.

Supplementary Figure S4. Alpha diversity indices of the microbial communities in the oil spill vicinity. A. Shannon index; B. Simpson index; C. Invsimpson index

Supplementary Figure S5. Phylogenetic tree assembled after shotgun sequencing representing MAGs taxonomy.

**Supplementary Methods**

***Method S1.*** *DNA extraction and metagenomic library preparation*

Total DNA extraction was performed using the phenol–chloroform protocol (Green and Sambrook 2017). Briefly, 20 mg/mL of lysozyme was added to the filter stored in lysis buffer and incubated at 37 °C for 30 min. Then, 20 mg/mL of proteinase K and a final volume of 20% SDS solution were added and incubated at 55 °C for 2 h. The lysate solution was transferred to a sterile 15 mL tube and one volume of phenol:chloroform:isoamyl alcohol (25:24:1, pH 7.7–8.3, Sigma-Aldrich, USA) was added. After 30 s of vortex, samples were centrifuged at 8000 × *g* for 10 min. The aqueous phase was transferred to a 15 ml tube for another step of phenol:chloroform:isoamyl alcohol 25:24:1. After further centrifugation at 8000 × *g* for 10 min, one volume of chloroform:isoamyl alcohol (24:1, Sigma-Aldrich, USA) was added to the retrieved aqueous phase. A centrifugation step at 8000 × *g* for 10 min was performed after a vortex step. The aqueous phase was transferred to a new 15 mL tube and DNA was precipitated at −20°C overnight, adding two volumes of ice-cold 100% absolute ethanol and 1/10 volume of sodium acetate (3.0 mol l^−1^ pH 5.3). DNA was then centrifuged for 30 min at 4°C and washed twice with ice-cold 80% ethanol. Ethanol was removed and DNA was resuspended in sterile 100 μL of Tris-HCl 10 mM pH 8.0. DNA was quantified with a Qubit® 3.0 Fluorometer using the Qubit® dsDNA HS assay kits (Thermo-Fisher Scientific) and DNA quality was assessed by gel electrophoresis with 1% agarose. The extracted DNA was stored at −20°C until library preparation.

16S rRNA libraries were prepared using the Illumina® Nextera XT Sample Prep Kit, following the Illumina® 16S rRNA metagenomic sequencing library preparation protocol. The amplicon targets the V3 and V4 variable region of bacterial 16S rRNA gene, using the following primer pair: Bac341F (CCTACGGGNGGCWGCAG) and Bac785R (GGATTAGATACCCVHGTAGTC) (Klindworth et al. 2012). The sequencing was performed by Illumina® MiSeq platform using the MiSeq reagent kit v3 600 cycles. Whole metagenome sequencing libraries were prepared using the Ovation Ultralow System V2 kit (NuGen, Tecan, Switzerland) following the manufacturer’s protocol. Sequencing was performed by Illumina® HiSeq4000 platform (2 x 150 bp cycle). All the sequencing was performed at the King Abdullah University of Science and Technology, Bioscience Core Lab (KAUST, BCL). The number of reads obtained from the 16S rDNA amplicon sequencing ranged between 90,000 and 150,000 reads per sample (Supplementary materials Fig. S1). Rarefaction curve for 16S amplicon sequencing for three surface water samples in the Red Sea is provided in Supplementary materials Fig. S2.

***Method S2.*** *Genome analysis*

Metagenome-Assembled Genomes (MAGs) were recovered following a modified version of a protocol used in Michoud et al. (Michoud et al. 2021). In brief, a combination of BinSanity v0.5.3 (Graham et al. 2017), Concoct v1.1 (Alneberg et al. 2014), Metabat2 v2.12.1 (Kang et al. 2019), and MaxBin2 v2.2.7 (Wu et al. 2015) were used in the binning step to reconstruct MAGs, and DasTool v1.1.2 (Sieber et al. 2018) was used to dereplicate and select non-redundant MAGs. Taxonomy was assigned using GTDB-toolkit v1.4.1 (Chaumeil et al. 2019), while the quality of the MAGs (completeness and contamination) was estimated with CheckM (v1.1.3) (Parks et al. 2015). We selected only MAGs with completeness higher than 70% and contamination lower than 10%. The coverage of each MAGs was assessed with CoverM (v0.6.1) (Woodcroft 2020) using the trimmed mean method. The coverage was normalized based on the abundance of the *recA* gene in each sample (Acinas et al. 2021). DESeq2 (Love et al. 2014) was used to assess significantly differential enriched MAGs in the communities across the sampling stations. Each MAGs was annotated with Bakta v1.1 (Schwengers et al. 2021), and EggNogMapper v.2.1.2 (Huerta-Cepas et al. 2018).

***Method S3.*** *Estimation of PAHs in zooplankton biomass*

PAHs in the zooplankton samples were analyzed using gas chromatography/tandem Triple Quadrupole mass spectrometry (GC-MS/MS) as described below. Firstly, the zooplankton samples were manually ground and homogenized in the presence of sand and anhydrous sodium sulfate. The homogenate was subsequently transferred to 33 mL pressurized liquid extraction (PLE) cells, which had been previously filled with 3 g of diatomaceous earth. Cells were extracted in a Dionex^TM^ ASE^TM^ Accelerated Solvent Extractor (Thermo Fisher scientific, Waltham, MA, USA) using dichloromethane as solvent. The optimum settings for PAH extraction were as follows: extraction temperature 100 ⁰C, static time 5 min, 2 cycles, purge for 60 s at 60% cell volume, purge with nitrogen and rinse with 10 mL of dichloromethane. The volume of the extract was approximately 35 mL. Prior concentration, 250 μL of 1-butanol were added to the extract as keeper. Extracts were concentrated down to 250 μL in a Genevac Rocket Evaporator (SP Industries, Warmister, PA, USA) for 60 min at 30 ⁰C under vacuum conditions. Extracts were reconstituted with 7 mL of dichloromethane and vortexed before removing the matrix interferences.

The above extracts were further cleaned up by gel permeation chromatography using a Robotic System FREESTYLE (LCTech Gmbh, Obertaukirchen, Germany). 5 mL of extract was injected to a 50 g Bio-Beads S-X3 column (66 x 44 m, ID = 25 mm). Dichloromethane at 5 mL/min was used as the mobile phase. The elution extract from 16.5 to 33.5 min was collected in an EVA evaporation module, where evaporation took place simultaneously under vacuum conditions. The eluate was then concentrated down to 2.5 ml using 480 mbar of vacuum. EVA cone heating was maintained at 35 ⁰C and the EVA water heating at 30 ⁰C. 900 μL of the final concentrate was transferred to a GC-vial to which 100 μL of internal standard was added. The clean extracts were analyzed by gas chromatography/tandem Triple Quadrupole mass spectrometry (GC-MS/MS, Agilent 7890 GC 7010B/MS) with a practical quantitation limit (PQL) of 0.01 μg L^−1^. One microliter of the extract was injected into a DB-EU PAH 20 m. 0.18 mm, 0.14 μm film thickness column. Quantification of total PAHs was done by an external calibration curve of PAHs ranging from 1 ng mL^−1^ to 100 ng mL^−1^ in dichloromethane with internal PAH standard at 100 ng mL^−1^. PAHs were confirmed by comparing retention times and ion ratios between sample extracts and standards, with a practical quantitation limit (PQL) of 0.04 μg L^−1^. The transitions used for PAHs determination are shown in Supplementary Information. This method included specific quality control parameters that are applicable to the US-EPA Method 8270 D (USEPA 1998).

**Supplementary Tables**

**Table S1.** Retention time (RT), internal standards (ISTD), transitions and collision energies (CE) used for the determination of PAHs.

| **Compound Name** | **RT^a^** | **Associated ISTD^b^** | **Transitions** | **CE (eV)** |
| --- | --- | --- | --- | --- |
| Naphthalene | 3.06 | Naphthalene-d_8_ | 128.0 > 102.0  128.0 > 127.0 | 22  20 |
| 2-Methylnaphthalene | 3.35 | Naphthalene-d_8_ | 142.0 > 115.0  142.0 > 141.0 | 30  30 |
| 1-Methylnaphthalene | 3.44 | Naphthalene-d_8_ | 142.0 > 115.0  142.0 > 141.0 | 30  30 |
| 2-Fluorobiphenyl | 3.55 | Acenaphthene-d_10_ | 172.0 > 169.9  172.0 >150.9 | 25  25 |
| Acenapthylene | 4.05 | Acenaphthene-d_10_ | 152.0 > 150.0  152.0 >150.9 | 40  40 |
| Acenapthene | 4.15 | Acenaphthene-d_10_ | 154.0 > 152.0  153.0 > 152.0 | 40  40 |
| Fluorene | 4.60 | Acenaphthene-d_10_ | 166.0 > 165.0  166.0 >163.0 | 30  33 |
| Phenanthrene | 6.23 | Phenanthrene-d_10_ | 178.1 > 176.0  178.1 > 152.1 | 34  25 |
| Anthracene | 6.32 | Phenanthrene-d_10_ | 178.1 > 176.0  178.1 > 152.1 | 34  25 |
| Fluoranthene | 9.98 | Phenanthrene-d_10_ | 202.0 > 201.0  202.0 > 200.0 | 50  50 |
| Pyrene | 11.22 | Chrysene-d_12_ | 202.0 > 201.0  202.0 >200.0 | 30  30 |
| 4-Terphenyl-d_14_ | 11.54 | 2,4-DDT-d_8_/ Chrysene-d_12_ | 244.0 > 242.2  244.0 > 240.2  244.0 >212.0 | 18  30  10 |
| Benz(a)anthracene | 18.01 | Chrysene-d_12_ | 228.0 > 226.0  228.0 > 226.0 | 38  38 |
| Chrysene | 18.52 | Chrysene-d_12_ | 228.0 > 226.0  228.0 > 226.0 | 38  38 |
| Benzo(b)fluoranthene | 25.33 | Perylene-d_12_ | 252.0 > 250.0  250.0 > 248.0 | 42  40 |
| Benzo(k)fluoranthene | 25.52 | Perylene-d_12_ | 252.0 > 250.0  250.0 > 248.0 | 42  40 |
| Benzo(a)pyrene | 27.97 | Perylene-d_12_ | 252.0 > 250.0  250.0 > 248.0 | 40  40 |
| Indeno(1,2,3,-cd)pyrene | 34.43 | Perylene-d_12_ | 276.0 > 274.0  276.0 > 247.0  276.0 > 276.0 | 42  60  15 |
| Dibenz(a,h)anthracene | 34.62 | Perylene-d_12_ | 278.0 > 276.0  278.0 > 252.0  278.0 > 278.0 | 38  60  15 |
| Benzo(g,h,i)perylene | 35.99 | Perylene-d_12_ | 274.0 > 272.0  277.0 > 275.0  276.0 > 274.0 | 42  38  42 |
| Naphthalene-d_8_ | 3.04 |  | 136.1 > 108.1  136.1 > 84.1 | 20  25 |
| Acenaphthene-d_10_ | 4.11 |  | 162.0 > 160.0  164.1 > 162.1 | 19  15 |
| Phenanthrene-d_10_ | 6.19 |  | 188.3 > 186.3  188.3 > 160.2 | 15  20 |
| Chrysene-d_12_ | 18.40 |  | 240.0 > 236.0  118.0 > 116.0 | 25  25 |
| Perylene-d_12_ | 28.61 |  | 264.0 > 260.0  264.0 > 236.0 | 40  25 |

^a^RT are indicative. They are subject to change depending on the column trimming

**Table S2**. Mean CO_2_, CH_4_, and δ^13^C values measured in seawater at the oil spill-affected Red Sea. NA, not analyzed.

| Location | CO_2_ ± SE  (ppm) | CH_4_ ± SE  (ppm) | δ^13^C-CO_2_ ± SE  (‰) | δ^13^C-CH_4_ ± SE  (‰) |
| --- | --- | --- | --- | --- |
| Station 1 | NA | NA | 0.88 ± 0.02 | NA |
| Station 2 | 201 ± 0.1 | 1.9 ± 0.0007 | −8.42 ± 0.02 | −48.7 ± 0.01 |
| Station 3 | 226 ± 0.15 | 1.91 ± 0.0004 | −7.77 ± 0.01 | −49.4 ± 0.02 |

**Table S3**. Variation in the MAGs among three sampling stations in the oil spill-affected Red Sea.

| **Station 1 vs Station 2** | | | |
| --- | --- | --- | --- |
| **MAGs** | **log2FoldChange** | **padj** | **Closest taxonomy** |
| HC_HiSeq_Metabat.104 | 2.4 | 3.70E-03 | g_Poseidonia |
| HC_HiSeq_Concoct.206_sub | 3.56 | 4.70E-03 | g_Rubripirellula |
| HC_HiSeq_Metabat.11 | 3.23 | 2.40E-02 | f_Opitutaceae |
| **Station 1 vs Station 3** | | | |
| **MAGs** | **log2FoldChange** | **padj** | **Closest taxonomy** |
| HC_HiSeq_Metabat.85 | 3.25 | 1.9E-05 | \| g_MGIIb-O5 \| \| --- \| |
| HC_HiSeq_Metabat.148 | 2.58 | 1.3E-04 | g_MGIIa-L1 |
| HC_HiSeq_Metabat.62 | 2.15 | 2.9E-03 | g_MGIIa-L2 |
| HC_HiSeq_Concoct.206_sub | 2.02 | 2.7E-02 | g_Rubripirellula |
| HC_HiSeq_Metabat.11 | 1.80 | 8.3E-03 | f_Opitutaceae |
| HC_HiSeq_Metabat.39 | 1.55 | 1.7E-02 | g_MGIIb-N1 |
| HC_HiSeq_Metabat.84 | 1.22 | 1.7E-02 | \| o_Pirellulales \| \| --- \| |
| HC_HiSeq_Metabat.22 | -1.02 | 7.9E-03 | f_UBA796 |
| HC_HiSeq_BinSanity_475 | -1.61 | 6.9E-03 | o_Phycisphaerales |
| HC_HiSeq_Metabat.140 | -4.04 | 1.7E-02 | g_Alcanivorax |
| HC_HiSeq_Metabat.176 | -10.22 | 2.4E-13 | g_Salinisphaera |
| HC_HiSeq_Metabat.99 | -13.58 | 1.3E-26 | g_Oleibacter |
| **Station 1 vs Station 3** | | | |
| **MAGs** | **log2FoldChange** | **padj** | **Closest taxonomy** |
| HC_HiSeq_Metabat.99 | -8.05 | 6.22E-27 | g_Oleibacter |
| HC_HiSeq_Metabat.176 | -9.89 | 2.40E-11 | g_Salinisphaera |
| HC_HiSeq_Metabat.140 | -3.65 | 1.94E-04 | g_Alcanivorax |
| HC_HiSeq_Concoct.209 | 3.49 | 1.32E-03 | g_Halomonas |

**Supplementary Figures**

**Figure S1**. 16S rRNA gene amplicon sequencing number of reads per samples.


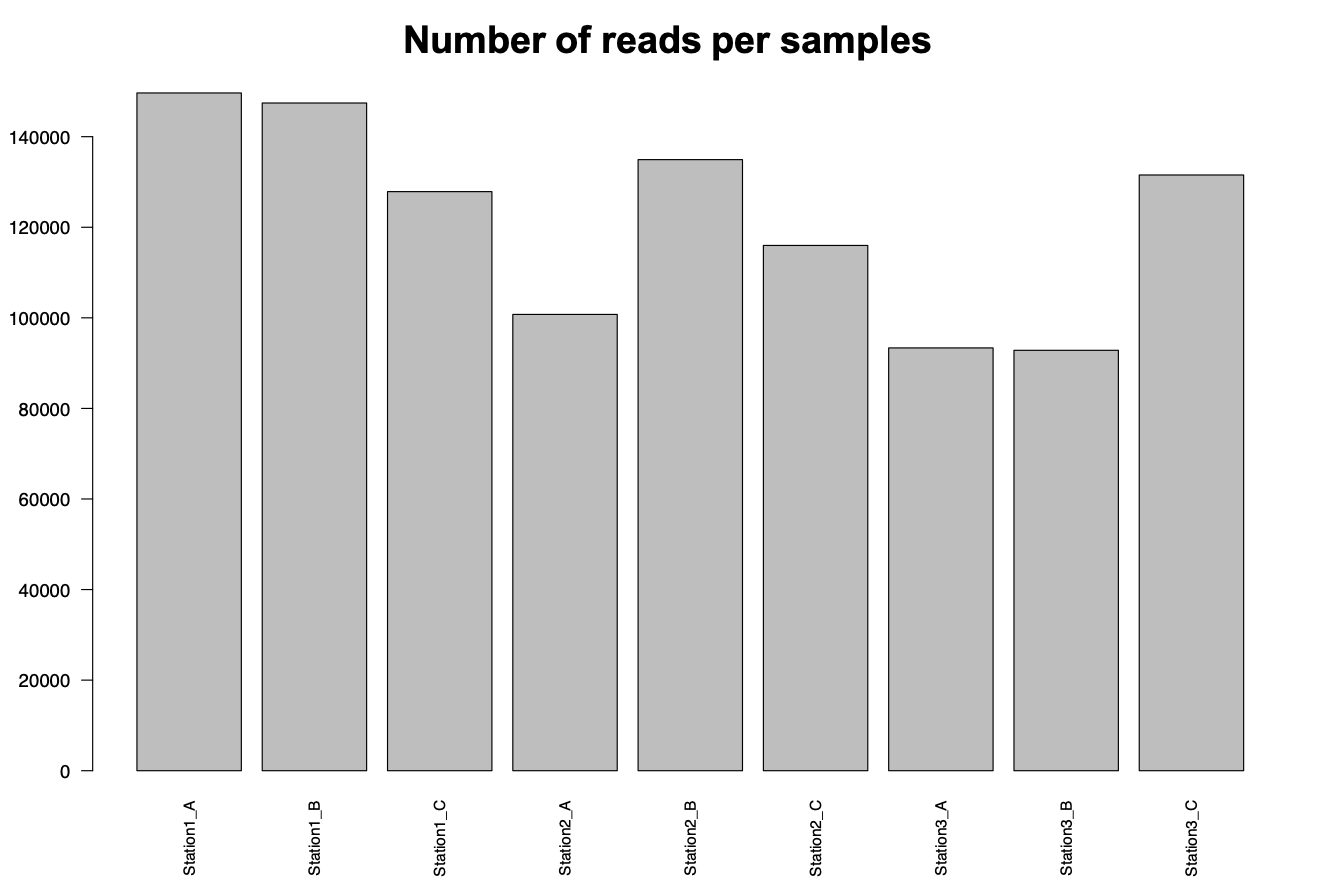


**Figure S2**. Rarefaction curve for samples at the oil spill site in the Red Sea.

**Figure S3**. Variation in major mineral levels in surface water in the oil spill-affected Red Sea.

**Figure S4**. Alpha diversity indices of the microbial communities in the oil spill vicinity. A. Shannon index; B. Simpson index; C. Invsimpson index.

A

B

**

C

**Figure S5**. Phylogenetic tree assembled after shotgun sequencing representing MAGs taxonomy.


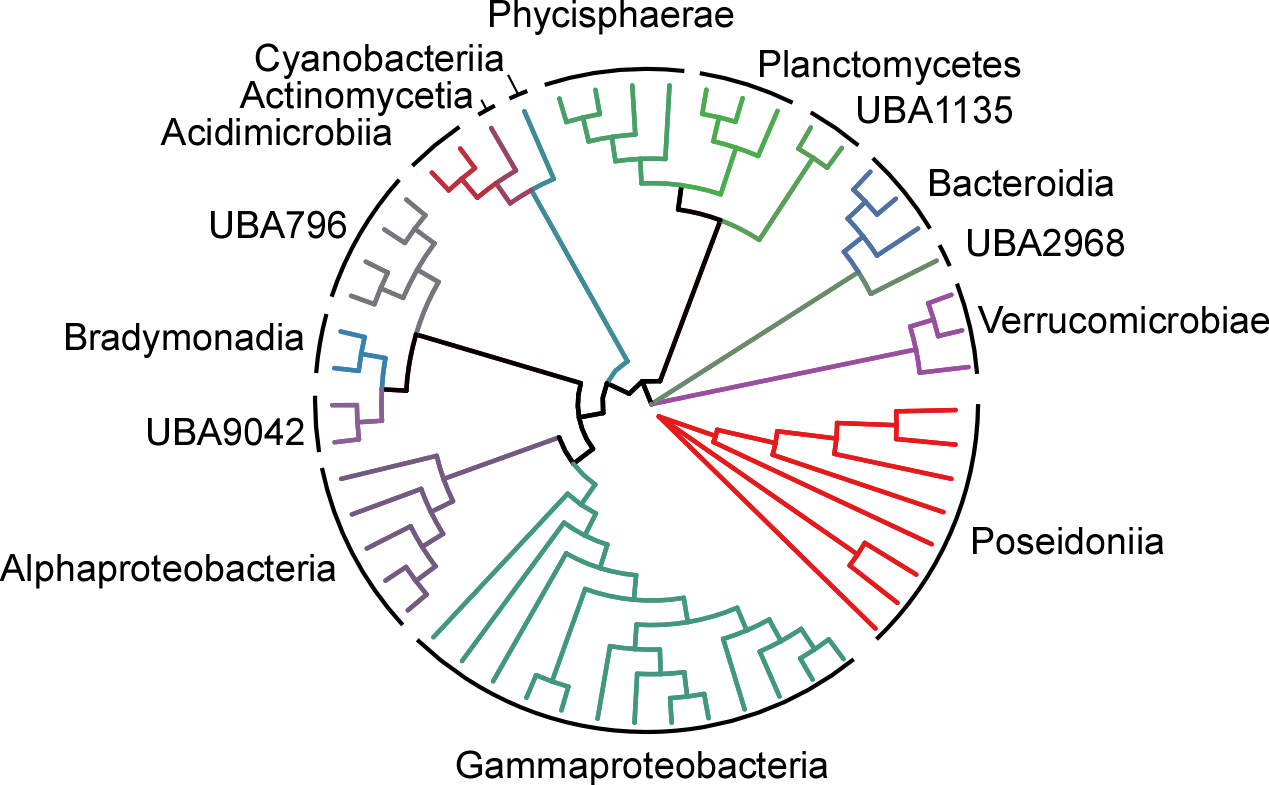


**References**

Acinas, S.G., Sánchez, P., Salazar, G., Cornejo-Castillo, F.M., Sebastián, M., Logares, R., Royo-Llonch, M., Paoli, L., Sunagawa, S., Hingamp, P., Ogata, H., Lima-Mendez, G., Roux, S., González, J.M., Arrieta, J.M., Alam, I.S., Kamau, A., Bowler, C., Raes, J., Pesant, S., Bork, P., Agustí, S., Gojobori, T., Vaqué, D., Sullivan, M.B., Pedrós-Alió, C., Massana, R., Duarte, C.M. and Gasol, J.M. (2021) Deep ocean metagenomes provide insight into the metabolic architecture of bathypelagic microbial communities. Communications Biology 4(1), 604.

Alneberg, J., Bjarnason, B.S., de Bruijn, I., Schirmer, M., Quick, J., Ijaz, U.Z., Lahti, L., Loman, N.J., Andersson, A.F. and Quince, C. (2014) Binning metagenomic contigs by coverage and composition. Nature Methods 11(11), 1144-1146.

Chaumeil, P.-A., Mussig, A.J., Hugenholtz, P. and Parks, D.H. (2019) GTDB-Tk: a toolkit to classify genomes with the Genome Taxonomy Database. Bioinformatics 36(6), 1925-1927.

Graham, E.D., Heidelberg, J.F. and Tully, B.J. (2017) BinSanity: unsupervised clustering of environmental microbial assemblies using coverage and affinity propagation. PeerJ 5, e3035.

Green, M.R. and Sambrook, J. (2017) Isolation of High-Molecular-Weight DNA Using Organic Solvents. 2017(4), pdb.prot093450.

Huerta-Cepas, J., Szklarczyk, D., Heller, D., Hernández-Plaza, A., Forslund, S.K., Cook, H., Mende, D.R., Letunic, I., Rattei, T., Jensen, Lars J., von Mering, C. and Bork, P. (2018) eggNOG 5.0: a hierarchical, functionally and phylogenetically annotated orthology resource based on 5090 organisms and 2502 viruses. Nucleic Acids Research 47(D1), D309-D314.

Kang, D.D., Li, F., Kirton, E., Thomas, A., Egan, R., An, H. and Wang, Z. (2019) MetaBAT 2: an adaptive binning algorithm for robust and efficient genome reconstruction from metagenome assemblies. PeerJ 7, e7359.

Klindworth, A., Pruesse, E., Schweer, T., Peplies, J., Quast, C., Horn, M. and Glöckner, F.O. (2012) Evaluation of general 16S ribosomal RNA gene PCR primers for classical and next-generation sequencing-based diversity studies. Nucleic Acids Research 41(1), e1-e1.

Love, M.I., Huber, W. and Anders, S. (2014) Moderated estimation of fold change and dispersion for RNA-seq data with DESeq2. Genome Biology 15(12), 550.

Michoud, G., Ngugi, D.K., Barozzi, A., Merlino, G., Calleja, M.L., Delgado-Huertas, A., Morán, X.A.G. and Daffonchio, D. (2021) Fine-scale metabolic discontinuity in a stratified prokaryote microbiome of a Red Sea deep halocline. The ISME Journal 15(8), 2351-2365.

Parks, D.H., Imelfort, M., Skennerton, C.T., Hugenholtz, P. and Tyson, G.W. (2015) CheckM: assessing the quality of microbial genomes recovered from isolates, single cells, and metagenomes. 25(7), 1043-1055.

Schwengers, O., Jelonek, L., Dieckmann, M.A., Beyvers, S., Blom, J. and Goesmann, A. (2021) Bakta: rapid and standardized annotation of bacterial genomes via alignment-free sequence identification. 7(11).

Sieber, C.M.K., Probst, A.J., Sharrar, A., Thomas, B.C., Hess, M., Tringe, S.G. and Banfield, J.F. (2018) Recovery of genomes from metagenomes via a dereplication, aggregation and scoring strategy. Nature Microbiology 3(7), 836-843.

USEPA (1998) Method 8270 D: semivolatile organic compounds by gas chromatography/mass spectrometry (GC/MS), US EPA Washington.

Woodcroft, B.J. (2020) ‘CoverM’. Available at: <https://github.com/wwood/CoverM>.

Wu, Y.-W., Simmons, B.A. and Singer, S.W. (2015) MaxBin 2.0: an automated binning algorithm to recover genomes from multiple metagenomic datasets. Bioinformatics 32(4), 605-607.
